# National Cancer Institute’s Cancer Disparities Research Partnership Program: Experience and Lessons Learned

**DOI:** 10.3389/fonc.2014.00303

**Published:** 2014-11-03

**Authors:** Rosemary S. L. Wong, Bhadrasain Vikram, Frank S. Govern, Daniel G. Petereit, Patrick D. Maguire, Maggie R. Clarkson, Dwight E. Heron, C. Norman Coleman

**Affiliations:** ^1^Radiation Research Program, Division of Cancer Treatment and Diagnosis, National Cancer Institute, Rockville, MD, USA; ^2^Walking Forward Program, Rapid City Regional Hospital, Rapid City, SD, USA; ^3^Coastal Carolina Radiation Oncology, Wilmington, NC, USA; ^4^Singing River Health System, Pascagoula, MS, USA; ^5^University of Pittsburgh Medical Center McKeesport, McKeesport, PA, USA

**Keywords:** cancer disparities, underserved populations, patient accrual, access to clinical trials, clinical research

## Abstract

**Purpose:** To increase access of underserved/health disparities communities to National Cancer Institute (NCI) clinical trials, the Radiation Research Program piloted a unique model – the Cancer Disparities Research Partnership (CDRP) program. CDRP targeted community hospitals with a limited past NCI funding history and provided funding to establish the infrastructure for their clinical research program.

**Methods:** Initially, 5-year planning phase funding was awarded to six CDRP institutions through a cooperative agreement (U56). Five were subsequently eligible to compete for 5-year implementation phase (U54) funding and three received a second award. Additionally, the NCI Center to Reduce Cancer Health Disparities supported their U56 patient navigation programs.

**Results:** Community-based hospitals with little or no clinical trials experience required at least a year to develop the infrastructure and establish community outreach/education and patient navigation programs before accrual to clinical trials could begin. Once established, CDRP sites increased their yearly patient accrual mainly to NCI-sponsored cooperative group trials (~60%) and Principal Investigator/mentor-initiated trials (~30%). The total number of patients accrued on all types of trials was 2,371, while 5,147 patients received navigation services.

**Conclusion:** Despite a historical gap in participation in clinical cancer research, underserved communities are willing/eager to participate. Since a limited number of cooperative group trials address locally advanced diseases seen in health disparities populations; this shortcoming needs to be rectified. Sustainability for these programs remains a challenge. Addressing these gaps through research and public health mechanisms may have an important impact on their health, scientific progress, and efforts to increase diversity in NCI clinical trials.

## Introduction

The Cancer Disparities Research Partnership (CDRP) pilot program was initiated by the radiation research program (RRP) within the National Cancer Institute (NCI)’s Division of Cancer Treatment and Diagnosis (DCTD) in 2002 as a novel strategy to address the cancer health disparities that exist in racial, ethnic, minority, and underserved populations within the United States (1). Over half of all cancer patients are treated with radiation alone or in combination with surgery or chemotherapy. This program was focused at radiation oncologists in community-based hospitals and cancer centers that predominantly serve minority/underserved populations. Since the goal was to reach populations not having access to NCI clinical trials, application criteria included limited participation in clinical trials, and NCI grant funding <$100,000/year. Utilizing a U56 planning cooperative agreement, funding went directly to community-based institutions to establish the clinical research infrastructure required for their populations to access NCI-sponsored radiation oncology-based clinical trials. CDRP sites were required to identify academic cancer centers or mentors experienced in NCI-sponsored clinical trials as partners who received limited funding from the grantee. To facilitate the mentoring relationships, a TELESYNERGY™ (2), telemedicine system was provided to both grantee and primary mentor. Furthermore, NCI’s Center to Reduce Cancer Health Disparities (CRCHD) provided supplemental funding to establish a patient navigation program addressing the specific needs of grantee’s targeted populations.

The primary goal was to increase accrual of minority/underserved populations into NCI-sponsored clinical trials. Other objectives were: (1) increasing the number of staff involved in cancer health disparities research; (2) assessing the value of implementing the TELESYNERGY™ telemedicine system; (3) implementing an appropriate patient navigation program; and (4) determining whether this pilot would work in community-based institutions not historically involved in clinical research.

## Materials and Methods

### Process for approval of CDRP concept initiative and reissuance

Following an NIH/NCI portfolio analysis, which determined a need for the proposed program, the CDRP concept was approved by the NCI’s Board of Scientific Advisors and the request for application (RFA-CA-02-002), was issued in October 2001. An NCI Special Emphasis Panel reviewed six applications and two awards were made in September 2002. Since initial funding was approved for up to six awards, RFA-CA-03-018 was issued in August 2002 and four additional awards were made out of eight reviewed applications producing a success rate of 43% from both RFAs. Table 1 includes information on the grantees, Principal Investigators (PIs), mentors, service areas, and target populations.

**Table 1:** **CDRP grantees, program title, PIs, mentors, service areas, and their target populations**.

Award year	Grantee/principal investigator (PI)	Service area population	Target population
FY02	Rapid City Regional Hospital, Rapid City, South Dakota Program name: Walking Forward (WF)^a^ PI: Daniel G. Petereit, MD Primary mentor: University of Wisconsin-Madison Secondary mentor: Mayo Clinic in Rochester, MN	300,000	American Indian/Native American
FY02	Laredo Medical Center^b^^;^ Laredo, Texas Program name: Evaluating Cancer Disparities Among Hispanic Communities PI: Yadvindera S. Bains, MD Primary mentor: University of Texas Health Science Center Secondary mentor: MD Anderson Cancer Center in Houston, TX	177,000	Hispanic/Latino
FY03	Daniel Freeman Memorial Hospital^c^Inglewood, CA, USAProgram name: Urban Latino African American Cancer (ULAAC) Disparities ProjectPI: Michael L. Steinberg, MD^d^Primary Mentor: University of Southern CaliforniaSecondary Mentor: RAND Corporation, Santa Monica, CA	100,000	African American Hispanic/Latino
FY03	New Hanover Regional Medical Center Wilmington, North Carolina Program name: Improving Cancer Outcomes for African-Americans PI: Patrick D. Maguire, MDPrimary mentor: University of North Carolina-Chapel Hill	616,000	African American Urban/Rural Poor
FY03	Singing River Hospital; Pascagoula, Mississippi Program name: The Mississippi/Alabama Radiation Oncology Research Partnership PI: Raymond Wynn, MD^e^ Primary mentor: University of Alabama at Birmingham Secondary mentor: University of Mississippi Medical Center	200,000	African American
FY03	University of Pittsburgh Medical Center (UPMC) McKeesport Hospital; McKeesport, Pennsylvania Program name: Radiation Oncology Community Outreach Group (ROCOG) PI: Dwight E. Heron, MD Primary mentor: Washington University, St. Louis, MO, USASecondary Mentor: Roswell Park Cancer Center, Buffalo, NY	935,000	African American Urban/Rural Poor

*^a^Rapid City changed its CDRP program name early in program development from “Enhancing Native American Participation in Radiation Therapy Trials” to “Walking Forward,” which was considered more culturally appropriate for their target American Indian patients*.

*^b^CDRP grant was relinquished in 2007 due to inability to find a qualified radiation oncologist to become PI when original PI resigned in 2006*.

*^c^Grant was changed to Centinela Freeman in 2004 and later was transitioned to Twenty-First Century Oncology at the Santa Monica Cancer Treatment Center in 2008*.

*^d^Dr. David Khan is the current CDRP PI and Dr. Michael Steinberg is co-PI*.

*^e^Dr. Raymond Wynn resigned in 2005 and Dr. W. Sam Dennis became the new PI*.

The formal process for reissuance of any currently funded NCI program changed in 2006 and required an external program evaluation. NOVA Research Company (3) was awarded the 5-year U56 CDRP Process and Outcome Evaluation contract, which helped generate the programmatic assessment data. The reissuance process used NOVA’s yearly CDRP Program Evaluation Reports (2006–2008) containing qualitative and quantitative data and the evaluation report by the CDRP Program Expert Committee (see Supplementary Material). This Expert Committee and CDRP PIs met annually at the American Society for Radiation Oncology (ASTRO) meeting to help RRP evaluate yearly progress and make recommendations for program improvement. The BSA recommended not expanding the program, but to accept applications only from the five funded grantees in a limited competition RFA-CA-09-502 (October 2008). After the Special Emphasis Panel review, 5-year U54 implementation awards were made to Rapid City Regional Hospital (RCRH), New Hanover Regional Medical Center (NHRMC), and Singing River Health System (SRHS), while UPMC McKeesport Hospital received a 2-year phase out award.

### Metrics of success

The metrics of success were:

1) Could a community-based hospital/cancer center establish a clinical research infrastructure within a reasonable period and accrue patients into radiation oncology-based clinical trials?
2) Was participation of underserved populations in NCI-sponsored clinical trials increased?
3) Were mentors helpful in providing necessary training, support, and advice to the grantees?
4) Was the TELESYNERGY™ telemedicine system beneficial to the CDRP programs?
5) Was the CDRP grantee successful in increasing the number of physicians/other staff interested in cancer disparities research?
6) Was the CDRP site successful in disseminating program results through publications/presentations at national meetings?

## Results

### Establishment of infrastructure

Establishing the clinical research infrastructure was challenging because institutions were unfamiliar with its value for their patients. Despite all PIs having prior clinical trials experience, it took many months to educate the hospital administration about the benefits for their patients by participating in NCI clinical trials. By offering trials near their hometowns, patients can access these clinical advances without traveling great distances.

Findings from the U56 pilot program revealed important and unique issues regarding outreach to disparities populations not encountered at academic cancer centers and their community oncology affiliates. Disparities researchers needed sufficient time to succeed in: (1) recruiting personnel due to challenges in finding qualified staff to fill positions (e.g., program/grant manager, clinical research nurse/coordinator, data manager, patient navigator, and regulatory affairs expert for writing clinical protocols); (2) identifying a back-up PI after the loss of the primary PI at Laredo Medical Center; (3) establishing an outreach program so the community gained familiarity and trust with the PI and the research team (4, 5); and (4) surveying the populations to determine their knowledge, attitudes, perceived barriers, and needs.

### Participation in clinical trials

After the infrastructure was established, there was steady patient accrual onto various NCI clinical trials (Table 2). The type of trials most useful to the grantees is discussed below. The fluctuation seen in patient accruals was due to the limited number of cooperative group trials available for minority/underserved populations presenting with late-stage disease and co-morbidities. The restrictive eligibility criteria for many cooperative group trials resulted in low eligibility rates, averaging only 20–24% during both U56 and U54 phases (Table 3) (6).

**Table 2:** **Cumulative number of patients accrued to different types of clinical trials by fiscal year in U56 planning phase and cumulative for U54 phase**.

Type of clinical trial	FY03^a^	FY04	FY05	FY06	FY07	FY08	FY09	Total U56 period^b^ (%)	Total U54 Period^c^ (%)
PI-initiated	0	1	44	78	78	38	62	301 (18)	139 (20)
Mentor-initiated	0	0	0	0	0	0	160	160 (10)	203 (29)
RTOG	10	7	17	26	34	24	50	168 (10)	128 (19)
Other cooperative groups^d^	271	349	39	75	84	98	82	998 (60)	209 (30)^d^
Radiation only	1	0	4	20	9	0	3	37 (4)	-
Radiation/combined treatment	65	9	14	24	19	13	14	158 (16)	-
Medical/surgical	140	25	24	27	55	79	57	407 (41)	*-*
Cancer control/prevention	75	316^e^	6	9	10	11	14	441 (44)	71 (45^f^)
Pharmaceutical/industry	0	0	5	1	8	6	31	51 (3)	14 (2)
Total	281^a^	357	105	180	204	166	385	1,678 (100^g^)	693 (100^g^)

*^a^Rapid City had approximately 33 active clinical protocols opened during FY03 in which they accrued 281 patients onto the STAR trial and cooperative group trials (RTOG and NCCTG) (*n* = 281)*.

*^b^U56 data are through September 30, 2009*.

*^c^Data cumulative for FY2010 through 2013 for all U54 grantees*.

*^d^Rapid City data include patients who were enrolled onto both RTOG and other cooperative group trials. The database structure at this site did not allow segregating the different trial categories (e.g., radiation only, medical/surgical) by only cooperative group trials. For U54 grantees, segregation was also not done*.

*^e^Includes Laredo’s accruals to NCI prevention trials, STAR (*n* = 9), and SABOR (300)*.

*^f^Percent accrual to prevention trials out of total NCI cooperative group trial accruals*.

*^g^Column percents do not total 100% due to rounding*.

**Table 3:** **Number of patients screened and eligible for cancer clinical trials**.

Grantee sites^a^	Patients screened		Patients eligible		Eligibility rate^b^ (%)	
	(U56)^c^	(U54)^d^	(U56)	(U54)	(U56)	(U54)
Rapid City	1,601	3180	457	340	29	11
Centinela Freeman	28	–	28	–	100^e^	–
New Hanover	228	2578	84	839	37	33
Singing River	982	2396	166	371	17	16
UPMC McKeesport	637	376	84	29	13^f^	8^f^
Total	3,476	8530	819	1579	24	20

*^a^Data were not available for Laredo*.

*^b^Eligibility rate is based on the number of patients eligible divided by the number of patients screened*.

*^c^U56 data are from FY07 through FY09 only*.

*^d^Data cumulative for FY2010 through 2013 for all U54 grantees*.

*^e^Centinela Freeman did not screen all patients*.

*^f^Only includes data on the UPMC McKeesport site out of a total of five participating hospital at this CDRP site*.

### Mentoring and partnership

All grantees selected an NCI-designated Comprehensive Cancer Center as a primary or secondary (Laredo) mentor (Table 1) based on clinical research expertise and/or an existing relationship; many also selected secondary partners to address specific needs. An important lesson learned was the need for the grantee to work immediately with the academic center’s grants research office to obtain details on job descriptions, guidance for establishing an Institutional Review Board (IRB), and assistance in grant management. Given the complexity of a clinical trials infrastructure, grantees required a year or more before their disparity program was fully operational leading some to restructure their awards to allow an additional year.

### Developing cancer disparities research interest and patient navigation

Because of the limited availability of cooperative group trials as noted above, the grantees used two approaches to expand protocol participation: (1) development of PI-initiated clinical trials targeting stage of disease and/or including shorter radiation therapy schedules to address patients’ transportation or accommodations barriers (7, 8) and (2) expanding access to other NCI-sponsored clinical trials beyond radiation oncology, to include surgical/medical oncology trials (Table 2). This expansion was facilitated starting in 2006 by Clinical Trial Operating Committee (CTOC) supplemental awards to RCRH, SRHS, and UPMC McKeesport for hiring clinical staff interacting with other oncology specialists and resulted in increased annual patient accruals (Table 2).

Having breadth in the portfolio of trials was critical to overall participation as shown in Table 2. Early on, prevention trials boosted accrual (FY03-04), but later on the increased accrual (FY06 onward) was due to availability of non-radiation trials. A total of 2,371 patients were accrued during both phases. The PI/mentor-initiated trials (see Supplementary Material) partially addressed the shortfall of trials suitable for this population as ineligibility remained an accrual barrier (Table 3).

In addition to informing patients about the clinical trials, the patient navigators supported by CRCHD had a positive influence on reducing the number of missed appointments and addressing other barriers to patient participation (9–11). Patient navigation was found to be a critical component at all CDRP sites as 5,147 patients were navigated (Table 4). RCRH and SRHS documented the critical benefit of patient navigators for their patients. Their data helped RCRH to receive a subsequent Komen Foundation grant specifically for a patient navigator to assist all their breast cancer patients, while SRHS’s patient navigator became a hospital funded position starting in 2011.

**Table 4:** **Number of navigated patients per fiscal year by CDRP grantee**.

Grantee sites	FY04	FY05	FY06	FY07	FY08	FY09	U56 total #^a^	U54 total #^b^
Rapid City	35	77	56	66	184	211	629	786
Laredo^c^	183	74	90	NA	NA	NA	347	NA
Centinela Freeman	NA^d^	80	146	166	90	127	609	NA
New Hanover	2	17	103	117	87	48	374	276
Singing River	NC	NR	NR	208	142	325	675	526
UPMC McKeesport	NA^d^	96	165	252	217	116	846	79
Total	220	344	560	809	720	827	3,480	1,667

*NA, not applicable; NC, data not collected by CDRP site; NR, refers to data not received*.

*^a^U56 data were consistently collected beginning in FY2007, Quarter 4 through September 30, 2009*.

*^b^Data cumulative for FY2010 through FY2013 for all U54 grantees*.

*^c^Laredo data are unconfirmed*.

*^d^Patient navigation program was not active until 2005*.

The American Indian (AI) Community Research Representatives (CRRs) were established with CDRP funding at three remote AI reservations in South Dakota. These trained community health educators and Patient Navigators helped RCRH receive a 2-year CDC grant by partnering with the South Dakota Health Department utilizing their CRRs to implement a colorectal screening program for their AI population. Additionally, RCRH partnered with the American Cancer Society to implement the “All Women Count!” Breast and Cervical Cancer Early Detection Program for their AI women at the Pine Ridge Indian Reservation whereby the goals of both ACS and RCRH’s *Walking Forward Program* were implemented (Dr. Petereit, personal communication, April 2014).

### TELESYNERGY™ – telemedicine and employment opportunities in remote communities

When the CDRP program was initiated, telemedicine was just being established and this program became a pilot test for TELESYNERGY™, a system developed jointly by the NIH Center for Information Technology and NCI. Table 5 details how it was used. Videoconferencing facilitated communication between awardees and their mentors for treatment planning (12) and follow-up consultations at remote settings (e.g., South Dakota and Pennsylvania). It was also used among CDRP sites predominantly for training clinical research staff, tumor board conferences, research consultations, and sharing of ideas. Establishing clinical consultation sites at remote centers resulted in saved patient travel time, and it also provided employment opportunities for healthcare workers on the reservations and at satellite Pennsylvania hospitals. These successes were important lessons learned from the conduct of clinical trials and medical care for remote disparities communities (12–14).

**Table 5:** Use of TELESYNERGY™ for CDRP grantee activities, by fiscal year – U56 phase*.

CDRP grantee activity	Number of times used for activity			
	FY07, Qtr 4	FY08	FY09	Total
Administrative meetings	5	29	28	62
Research consultations	21	8	16	45
Patient consultations	612	2,037	237	2,886^a^
Tumor boards	12	113	108	233
Training/education	5	10	20	35
Other^b^	8	4	14	26
Total	663	2,201	423	3,287

*Data were consistently collected beginning in FY2007, quarter 4 through September 30, 2009.

*^a^99% of the patient consultations via TELESYNERGY^®^ were conducted by Rapid City*.

*^b^Includes TELESYNERGY^®^ maintenance and patient rounds*.

### Dissemination of results

The CDRP institutions were very active in presentations at local meetings and nationally at the Radiation Therapy Oncology Group (now part of NRG Oncology) and the annual ASTRO meetings. Additionally, the CDRP program helped establish an annual ASTRO/NCI Cancer Disparity Symposium to help educate members about cancer disparities issues in the U.S. (see Supplementary Material). All CDRP sites were active to various degrees in publishing results of their cancer disparities program with RCRH being the most productive with publications (Table 6).

**Table 6:** **Number of CDRP-related publications by grantee site**.

Grantee	Number of publications	
	U56	U54
Laredo	1	NA
Rapid City	23^a^	24
Centinela Freeman	8	NA
New Hanover	4	4
Singing River	3	0
UPMC McKeesport	14	3
Total	53	31

*^a^Includes three book chapters*.

## Discussion

As pointed out in a BSA discussion, CDRP took on some of the most difficult challenges to develop clinical trials because of both the disparity populations and the limited previous NIH funding history. Although a higher level of trial participation may have been possible with health disparities sites within the catchment of an NCI-designated Cancer Center and the Division of Cancer Prevention’s Minority-Based Community Clinical Oncology Program site, that CDRP successfully reached into the more difficult-to-reach areas dispelled the concept that this was impossible and/or that people would reject participation in trials.

To expect health disparities sites to achieve similar rates of clinical trial accrual as major cancer centers and their catchment area is not realistic. Implementation takes time and include: (1) developing/training personnel with the necessary research skills and staff to work with government regulations for clinical trials; (2) advising administrators and hospital leadership about the patience needed to develop infrastructure and the wisdom to see the benefit to patients and institution; and (3) establishing physical space and technological facilities needed to conduct research and manage the data.

Having an experienced team from NCI initiate the programs with a visit to the institution was also important. Although there was skepticism based on the experiences that the government would “establish a program, do research, and then leave (14),” this support helped establish trust and personal relationships demonstrating that the government was invested in the community’s problems. The initial NCI team included physicians, senior administrators, program directors, and a patient advocate provided by the National Coalition for Cancer Survivorship (15) who emphasized the central importance of community buy-in at the outset.

We suggest that the proposed metrics of success for future disparities efforts include the usual “hard” metrics such as clinical trial participation and publications, but also softer metrics such as: (1) surveys conducted, (2) the number of patients screened, (3) the extent of outreach–recruitment activity, (4) additional research efforts leveraging their infrastructure, (5) staff recruitment, (6) enhanced interest in disparities by cooperative groups (e.g., RTOG) and professional societies (ASTRO), and (7) the ability to secure additional funding. Formal program evaluation as established by NOVA was extraordinarily helpful for the awardees and RRP in assessing progress and determining both gaps and opportunities for progress. The CDRP programs shared data and trials among the awardees, which facilitated the implementation science.

Several years are needed to ramp up clinical trial participation, including the need for surveys and focus groups, time to listen to the community, understanding their needs, assessing barriers and building teams and trust (5, 16). Establishing trusting partnerships with the AI community in SD was a potential barrier for the Walking Forward (WF) program that was successfully addressed over time and became evident when there was no difficulty in consenting patients to participate in the ATM genetic study (17). For the advanced stage diseases encountered in minority/underserved populations and limited number of cooperative group studies available, designing PI- and mentor-initiated trials and the later expansion to a broader portfolio of trials beyond radiation oncology resulted in increased accrual for these patient populations (Table 2).

Telemedicine has evolved significantly as the TELESYNERGY™ system progressed from expensive ISDN phone line systems to a web-based system using off-the-shelf technology. A simplified version is now available for public purchase by outside institutions, both nationally and internationally, with the possibility for support for technical consultations, installation, and training from NIH/NCI if needed.

The establishment of CDRP led to some important changes in the radiation oncology community. The RTOG (now part of NRG Oncology) raised the issue of the need for future “health disparities” clinical trials to fill the gaps noted above. ASTRO developed an annual health disparities scientific program (see Supplementary Material) and additional radiation oncology activities related to cancer disparities on the international level include working with the Program of Action for Cancer Therapy (PACT) of the International Atomic Energy Agency (IAEA) (18)

Perhaps, the greatest challenge now is program sustainability. There were positive outcomes that were not predicted and are strong evidence of the value of investing in NIH/NCI clinical research in health disparities regions. Examples are provided from the three 10-year awardees.

- Rapid City Regional Hospital, in addition to coordinating the state programs mentioned previously, established collaborations with the Native American Cancer Research Corporation (NACR-PI Linda Burhansstipanov, DrPH), the University of Washington-Seattle (Dedra Buchwald, MD), Marquette University (Sheikh Iqbal Ahamed, PhD), and the University of New Mexico (Emily Haozous, PhD) to increase cancer screening and palliative care programs in the remote reservations for AIs. In addition, two resident physicians from Harvard University performed their research year at RCRH: Ashleigh Guadagnolo, MD, MPH (radiation oncology) and Sunshine Dwojak, MD (head and neck surgery). RCRH was awarded a 4-year NCI Provocative Questions R01 grant in 2012 by leveraging their CRRs to implement an “*American Indian mHealth Smoking Dependency Study*” program for the AIs in South Dakota. Several publications resulted from these collaborations with the WFP that ultimately assisted with program sustainability (4–6, 19–21).
- Coastal Carolina Radiation Oncology (CCRO) used their CDRP success in patient accrual to become a full member of NRG Oncology. To sustain their disparity program, CCRO again partnered with NHRMC to form the Southeastern North Carolina (SENC) CCOP, a grant that was awarded in 2013. Because the minority population in SENC is <50% of the population, the SENC CCOP could not apply to become a new NCI’s Community Oncology Research Program (NCORP) Minority/Underserved Community Site in 2014. Instead, CCRO will revert to full membership in NRG, while NHRMC will likely be an affiliate member of the Alliance group.
- Singing River Health System is building on their mentor relationship with University of Alabama Birmingham (UAB) Cancer Center. It will expand their current patient navigation program via participation as the only Mississippi-based site participating in the CMS Health Care Innovation Challenge award – *Deep South Cancer Navigation Network* and will receive $1 million over 3 years to support 3.5 FTEs for their patient navigation Program.

Philanthropy is another means of potential support, but community resources that major cancer centers or cooperative groups have are not accessible in these health disparities communities. For them to raise sufficient funding to sustain their infrastructure and professional staff and become competitive for the major infrastructure, clinical trials and center grants available are not a reasonable expectation.

## Conclusion

Through the CDRP program, clinical trials were established in health disparities sites not previously participating in the NCI clinical trials enterprise. The initial success of this pilot program is reassuring and may lead to improved general healthcare awareness for their minority/underserved populations and an increase in the diversity in NCI clinical trials.

Health disparity is an economic issue as much as a “minority” issue. Some health disparities regions have unique populations (i.e., the AIs) and when the study of the biological basis of cancer is conducted for their benefit, trust can be established and “precision/personalized” medicine targeting their illnesses can then be investigated.

The BSA review of the program renewal raised the issue of moral obligation for sustaining programs. Federal agencies support all of the people and the CRCHD has emphasized the large potential value of applying what we already know to help improve cancer outcomes for health disparities communities. While some of what CDRP accomplished met the standard metrics of clinical research as judged by participation numbers, there are aspects that were indeed unique and fall within implementation science. Lessons learned are applicable to future programs. In regard to the moral issue, research programs must pass peer review but, perhaps, different metrics are justified for the disparities sites that suffer from a lack of infrastructure and experience when competing for grants. For all the sites, especially the three 10-year awardees, the CDRP program succeeded in bringing people to clinical trials who previously were on the periphery and without access to the potential advances from these studies. This pilot program showed that reaching health disparities communities who are new to cancer research can be done and the future challenge is not only to broaden access to appropriate cancer care for health disparities populations, but also to sustain these gains.

## Conflict of Interest Statement

The authors declare that the research was conducted in the absence of any commercial or financial relationships that could be construed as a potential conflict of interest.

## Supplementary Material

The Supplementary Material for this article can be found online at http://www.frontiersin.org/Journal/10.3389/fonc.2014.00303/abstract

## Abbreviations

ACS, American Cancer Society; AI, American Indian; ASTRO, American Society for Radiation Oncology; ATM, ataxia telangiectasia mutated gene; BSA, Board of Scientific Advisors for National Cancer Institute; CCRO, Coastal Carolina Radiation Oncology; CCOP, Community Clinical Oncology Program; CDRP, Cancer Disparities Research Partnership; CRCHD, Center to Reduce Cancer Health Disparities; CRR, Community Research Representative; CTOC, Clinical Trials Operating Committee; DCTD, Division of Cancer Treatment and Diagnosis; IAEA, International Atomic Energy Agency; IRB, Institutional Review Board; MB-CCOP, Minority-Based Community Clinical Oncology Program; NACR, Native American Cancer Research; NCI, National Cancer Institute; NCORP, National Cancer Institute Community Oncology Research Program; NHRMC, New Hannover Regional Medical Center; NIH, National Institute of Health; PACT, Program of Action for Cancer Therapy; PI, principal investigator; RCRH, Rapid City Regional Hospital; RFA, request for application; RRP, Radiation Research Program; RTOG, radiation therapy oncology group; SENC, Southeastern North Carolina; SRHS, Singing River Health System; UAB, University of Alabama Birmingham; UPMC, University of Pittsburgh Medical Center; WFP, Walking Forward Program.
