# Supplementary material for: National Cancer Institute’s Cancer Disparities Research Partnership Program: Experience and Lessons Learned
Source: Front Oncol. 2014 Nov 3;4:303. doi: 10.3389/fonc.2014.00303 (PMC4217306; doi:10.3389/fonc.2014.00303)
Supplement: Supplementary file 1 [file Data_Sheet_1.PDF]

Appendix 1

Cancer Disparities Research Partnership (CDRP) Program
Program Expert Committee

1. ***Debra Thaler-DeMers, RN, OCN***
   Ms. Thaler-DeMers is a clinical staff nurse, ONS Chemotherapy and Biotherapy Instructor and End of Life Nursing Education Curriculum Instructor at the Petersen Cancer Treatment Center, Stanford University Hospital and Clinics. Debra is the founder of Cancer ACCESS: Advocacy, Counseling, Clinical Education & Survivorship Skills. She is a certified oncology nurse, a pain resource nurse and a specialist in end of life care. Debra is a twenty-two year survivor of two primary cancers and has served on the Board of Directors of the National Coalition for Cancer Survivorship. She is very knowledgeable and well informed about the range of issues the CDRP is dealing with especially with disparity populations. Ms. Thaler-DeMers lost her battle with cancer on January 13, 2010.

2. ***Kathryn M. Kash, PhD***
   Dr. Kash is Associate Professor of Psychiatry and Human Behavior at Thomas Jefferson University/Jefferson Medical College. She completed her graduate work in Psychology at the Graduate School and University Center – City University of New York and completed her fellowship at Memorial Sloan-Kettering Cancer Center. She has focused on helping young women with breast cancer cope with the disease and its impact on their lives. Dr. Kash's clinical practice is in the treatment of the emotional distress associated with cancer, primarily women with breast cancer and helps women make informed decisions about genetic testing for cancer susceptibility, treatment for breast cancer, and strategies for the prevention of breast and ovarian cancer. Her current research interests are looking at how women make decisions about their health care, complimentary approaches to breast cancer treatment and determining patient preferences for enrolling in clinical trials.

3. ***Carl M. Mansfield, MD, ScD, FACR, FACNM***
   Dr. Mansfield is retired, but was the Associate Director of the Greenbaum Cancer Center, University of Maryland Medical System in addition to Professor and Chairman of the Radiation Oncology Department at the University of Maryland Medical Center until 2002. His research has been in the treatment of cancer focusing on breast cancer. From 1967 to 1983, Dr. Mansfield was Professor and Chairman of the Department of Radiation Oncology at Thomas Jefferson University Hospital. From 1994 through 1997, he was Associate Director of the Radiation Research Program at the National Cancer Institute. Dr. Mansfield was an early advocate of treating breast cancer patients with lumpectomy instead of mastectomy.

Appendix 2

PI-Initiated Radiation Oncology Clinical Trials

Rapid City Regional Hospital

- A Pilot Phase I/II Study of Hypo-Fractionated External Beam Radiation and HDR Brachytherapy for Advanced Prostate Cancer (U56)
- A Phase II Trial to Evaluate HDR Brachytherapy as Monotherapy for Stage I and II Breast Carcinoma (U56)
- Phase I/II Trial Examining Dose-Per-Fraction Escalation Using Helical Tomotherapy in the Treatment of Prostate Cancer (U56)
- Ataxia Telangiectasia Mutation (ATM) Variant in Native Americans: Possible Association with Cancer and Radiotherapy Toxicities (U56)
- A Phase II Prostate Cancer Trial: High Risk Prostate Intensity Modulated Radiation Therapy (IMRT) for the Treatment of Pelvic Lymph Nodes and the Prostate to High Dose (U56)
- Cancer Patient Navigation (U56 and U54)
- Retrospective, Match Paired Analysis of Locally Advanced Head and Neck Patients Treated With Conventional Radiation (XRT) vs Intensity-Modulated Radiotherapy (IMRT)(U54)
- Community Assessment of Palliative Care Needs and Knowledge (U54)
- Treatment, Outcomes and HPV Status Among American Indians in South Dakota with Head and Neck Cancer (U54)
- Knowledge of Risk Factors and Early Symptoms of Head and Neck Cancer Among American Indians in South Dakota (U54)
- Walking Forward Navigator-Driven Community Education and Screening (U54)

New Hanover Regional Medical Center

- Phase II Trial of Hyperfractionated Intensity Modulated Radiotherapy (HMRT) with Concurrent Weekly Cisplatin for Stage III and IVA Head and Neck Cancer (U56)
- The Influence of Cognitive and Psychosocial Factors on Cancer Treatment (U56)
- Evaluation of Improving Cancer Outcomes for African-Americans (Navigation) (U56)
- Phase II Evaluation of Hypofractionated Breast Irradiation in Patients Undergoing Standard Lumpectomy or Oncolytic Mammoplasty with Bilateral Breast Reduction (U54)
- Chemoradiotherapy with Very Low Dose Elective Nodal IMRT for Locally Advanced Head and Neck Cancer: A Multi-institutional Phase II Study (U54)
- Multicenter Randomized Phase II Study of Erlotinib, Cisplatin and Radiotherapy versus Cisplatin and Radiotherapy in Patients with Stage III and IV Squamous Cell Carcinoma of the Head and Neck (Mentor-Initiated – U54)
- A Randomized Study to Determine Whether ArginMax Improves Sexual Function and Quality of Life in Female Cancer Survivors (Mentor-Initiated – U54)
- Randomized Study of Soy and Effexor Vasomotor Symptoms of Men Receiving Hormonal Therapy for Prostate Cancer (Mentor-Initiated – U54)
- Phase II Study of Stereotactic Body Radiation Therapy (SBRT) after First-Line Chemotherapy for Metastatic NSCLC (Mentor-Initiated – U54)
- A Phase II Study of Stereotactic Body Radiotherapy (SBRT) for Prostate Cancer Using Continuous Real-Time Evaluation of Prostate Motion and IMRT Plan Reoptimization Based on Same Day Anatomy (Mentor-Initiated – U54)
- Impact of Genomics and Exposures on Disparities in Breast Cancer (Mentor-Initiated – U54)

Appendix 3

ASTRO/NCI Cancer Disparity Symposia and Topics
Since CDRP Program Started in September 2002

45th ASTRO Annual Meeting in Salt Lake City, UT on Oct. 19-23, 2003

Tuesday, Oct. 21, 2003 – ASTRO/NCI Cancer Disparity Symposium & Reception

Bringing Diversity to Radiation Oncology

Dr. Wallner/Dr. Govern – RRP Initiatives; Drs. Petereit and Bains – CDRP Projects; Ms. Daly – ASTRO Disparities Initiatives and Dr. Trimble – CTEP Disparities Initiatives

46th ASTRO Annual Meeting in Atlanta, GA on Oct. 3-7, 2004

Wednesday, Oct. 6, 2004 - ASTRO/NCI Cancer Disparity Symposium & Reception

Developing Radiation Oncology Models to Address Cancer Disparities

Keynote Speaker - Dr. Otis W. Brawley

47th ASTRO Annual Meeting in Denver, CO on Oct. 16-20, 2005

Sunday, Oct. 16, 2005 - ASTRO/NCI Cancer Disparity Symposium & Reception

Patient Navigation: Facing the Challenge of Involving Underserved Populations in Clinical Trials

Keynote Speaker – Dr. Harold Freeman

48th ASTRO Annual Meeting in Philadelphia, PA on Nov. 5-9, 2006

Monday, Nov. 6, 2006 - ASTRO/NCI Cancer Disparity Symposium & Reception

Do Navigators Make a Difference in Acceptance of Clinical Trials?

Michael Steinberg, MD; PI ULAAC CDRP Program, Inglewood, CA

The Cancer Disparities Research Partnership Program Experience in Southern Mississippi

W. Sam Dennis, MD; PI of Singing River Hospital CDRP Program, Pascagoula, MS

49th ASTRO ANNUAL Meeting in Los Angeles, CA on Oct. 28 – Nov. 1, 2007

Monday, Oct. 29, 2007 - ASTRO/NCI Cancer Disparity Symposium & Reception

Accrual Strategies to Clinical Trials

David G. Brachman, MD - Arizona Oncology Services & Foundation

William J. Hicks, MD – Ohio State University

50th ASTRO Annual Meeting in Boston, MA on Sept. 21-25, 2008

Monday, Sept. 22, 2008 – ASTRO/NCI Cancer Disparity Symposium & Reception

Cancer Disparities Research: Sustaining a National Priority

Timothy Kinsella, MD

Center to Reduce Cancer Health Disparities – Program Overview

Martha Hare, PhD, RN: Program Director-CRCHD

51st ASTRO Annual Meeting in Chicago, IL on Nov. 1-5, 2009

Monday, Nov. 2, 2009 - ASTRO/NCI Cancer Disparity Symposium & Reception

A Multifaceted Approach to Lower Cancer Mortality Rates Among American Indians

Daniel G. Petereit, MD – PI of Rapid City Regional Hospital CDRP Program

52nd ASTRO Annual Meeting in San Diego, CA on Oct. 31 – Nov. 4, 2010

Monday, Nov. 1, 2010 - ASTRO/NCI Cancer Disparity Symposium & Reception

New Hanover Regional Medical Center: Community Cancer Center Succeeds in Providing Access to NCI Cancer Clinical Trials for Their Southeastern North Carolina Patients

Patrick D. Maguire, MD – PI of New Hanover Regional Medical Center CDRP Program

The Role of Patient Navigation and Community Outreach in a Successful Cancer Disparity Research Partnership (CDRP) Program

Maggie R. Clarkson, RN, MS, Program Director of Singing River Health System CDRP Program

53rd ASTRO Annual Meeting in Miami Beach, FL on Oct. 2-6, 2011

Monday, Oct. 3, 2011 - ASTRO/NCI Cancer Disparity Symposium & Reception

The Challenges Ahead in Increasing Access to Clinical Trials for the Elderly

Martine Extermann, MD, MPH; H. Lee Moffitt Cancer Center, Tampa, FL.

The Impact of 2011 Medicare Changes on Participation of the Elderly in Clinical Trials

Dwight E. Heron, MD, PI of UPMC McKeesport Hospital CDRP Program

54th ASTRO Annual Meeting in Boston, MA on Oct. 28-31, 2012

Monday, October 29, 2012 - ASTRO/NCI Cancer Disparity Symposium & Reception

Improving Cancer Care Globally: A Call to Service in Radiation Oncology

CANCELLED Due to Super Storm Sandy

55th ASTRO Annual Meeting in Atlanta, GA on September 22-26, 2013

Monday, September 23, 2013 - ASTRO/NCI Cancer Disparity Symposium & Reception

Improving Cancer Care Globally: A Call to Service in Radiation Oncology

ASTRO-ARRO Resident Experience in the Global Health Scholarship Program

Nina Mayr, MD

Desperate and Disparate in a Time of Many Transitions: Improving Research and Practice in Radiation Oncology ----

Glenn Jones, MD

Possible Business Models for Radiation Therapy Facilities in Bangladesh

Richard Love, MD
